# Achievable Rate Maximization for Multi-Relay AF Cooperative SWIPT Systems with a Nonlinear EH Model

**DOI:** 10.3390/s22083041

**Published:** 2022-04-15

**Authors:** Yizhi Feng, Yan Cao

**Affiliations:** 1 School of Electronic and Information Engineering, South China University of Technology, Guangzhou 510640, China; eeyancao@scut.edu.cn; 2 The National Engineering Technology Research Center for Mobile Ultrasonic Detection, South China University of Technology, Guangzhou 510640, China

**Keywords:** nonlinear energy harvesting, cooperative communication, multiple relays, achievable rate maximization

## Abstract

In this paper, the maximization of the achievable information rate is proposed for the multi-relay amplify-and-forward cooperative simultaneous wireless information and power transfer communication systems, where the nonlinear characteristic of the energy harvesting (EH) circuits is taken into account for the receivers of the relay nodes. The time switching (TS) and power splitting (PS) schemes are considered for the EH receivers and the achievable rate maximization problems are formulated as convex and non-convex optimization problems, respectively. The optimal TS and PS ratios for the relay nodes along with the maximum achievable rates for the system are obtained, respectively, by solving the optimal problems with efficient algorithms. The asymptotic maximum achievable rates at low and high input signal-to-noise ratios (SNRs) for both the PS and TS schemes are also analyzed. It is demonstrated that the PS scheme is more susceptible to the variation of the relays’ location and the channel parameters than TS scheme, whereas the TS scheme is more susceptible to the mismatch of the resource allocation than PS scheme. Specifically, compared to the linear EH model, the nonlinear EH model achieves significant performance gain for the TS scheme, whereas inconspicuous performance improvement is achieved for the PS scheme.

## 1. Introduction

Radio frequency energy harvesting (RFEH) is a very attractive and promising solution to prolonging the lifetime for low-power and energy-limited wireless devices and networks, such as wireless sensor networks, wireless body area network, and Internet of Things (IoT) [1]. By harvesting energy from the comparably controllable and human-made radio frequency (RF) signals, the RFEH technique offers on-demand and stable energy supplies and, thus, introduces predictable and reliable self-sustainability to the energy-constrained wireless device [2,3]. As one of the most attractive RFEH techniques, simultaneous wireless information and power transfer (SWIPT) can realize utilization of the RF signals for both wireless power transfer (WPT) and wireless information transfer (WIT) simultaneously, and thus, support the applications with the requirements of quality-of-service (Qos) [1,4]. With the unified design of WIT and WPT, SWIPT can achieve optimal resource allocation and compromise between WIT and WPT, thus making the best use of the network infrastructure and the RF radiation/spectrum [3]. SWIPT has attracted a growing interest and has become appealing in the wireless communication area [3,4,5,6,7,8,9,10]

### 1.1. Related Work

SWIPT has been studied for wireless cooperative systems in [11,12,13,14,15,16,17,18,19], where the energy-limited relay nodes are capable of harvesting energy from the received source’s RF signals. Specifically, the cooperative amplify-and-forward (AF) wireless networks were considered for the performance analysis in [11,12], where the bit-error-rate (BER) performance was studied for the systems with differential modulation in [11] and the outage probability and BER performance was studied for the systems with both coherent and non-coherent modulations in [12]. In [13,14,15], the decode-and-forward (DF) protocol was applied to the relaying systems, where the long-term throughput was investigated in [13] for buff-aided hybrid relay, while the outage probability was analyzed in [14,15], respectively, for a single source-destination pair with spatially random relays and the non-orthogonal multiple access (NOMA). In [16], the AF relay systems with an adaptive receiving architecture for information processing and energy harvesting (EH) were considered, where the ergodic capacity and the outage performance were derived. Besides a performance study for the SWIPT cooperative system with different relay protocols, various resource allocation schemes were proposed to enhance the system performance. In particular, to maximize the system throughput, the authors in [17] considered AF relay networks and proposed the joint relay selection and power allocation schemes, and in [18], the authors proposed the beamforming design for full-duplex multiple-input single-output relay systems. In [19], a joint optimization scheme of relay placement, power splitting (PS), and power allocation was proposed to improve the outage performance for the DF relay systems. The joint EH scheduling and beamforming was proposed in [20] for the SWIPT systems with multiple relays working in a time switching (TS) mode. In [21], the power splitting ratios at multiple relays were optimized for maximizing the information rate for the relaying systems with DF and AF protocols. In [22,23], the outage and throughput performance, and the joint user paring and resource allocation were, respectively, presented for the SWIPT-enabled cooperative NOMA systems.

The aforementioned papers considered the simple and ideal linear EH model, where linear input-output characteristic was assumed for the energy harvester at the receiver. However, practical measurement and observation has shown the nonlinearity of the input-output characteristic of practical energy harvester [3,24]. Recently, the nonlinear EH model has attracted increasing interest in the SWIPT system design and performance analysis. In [25], a parametric and circuit-specific nonlinear EH model was presented based on the curve fitting method. Using this parametric nonlinear EH model, [26] proposed a joint power control and time allocation scheme for the MIMO SWIPT systems to maximize the system throughput, while [27] explored the rate-energy region for the SWIPT system in the MIMO broadcasting channel, and [28] proposed a jointly optimal design of the PS ratios, artificial noise covariance, and transmission precoding matrix to optimize the secrecy energy efficiency of the secure SWIPT systems. Another nonlinear EH model described by piece-wise linear function was considered in [29,30,31], respectively, for the outage capacity calculation of AF relaying systems, the outage performance analysis of DF relaying systems, and the BER expressions derivation and diversity order analysis of AF relaying systems.

### 1.2. Motivation and Contributions

Except that a few studies (e.g., [14,20,21], etc.) considered multiple relays, the aforementioned literature on SWIPT relay systems focused on a single relay model. However, the power harvested at a single relay is typically small and a single relay may not provide satisfactory cooperative quality for the transmission [20]. Moreover, most of the works on SWIPT relay systems used linear EH model in the system design or performance analysis, while bringing the benefit of easy and tractable analysis, the use of conventional linear EH model for system design in the SWIPT systems may lead to the mismatches of resource allocation and thus resulting in the performance degradation, since the linear EH model is usually inaccurate and less practical in modeling the realistic nonlinear EH implementations [25,26].

Motivated by the above observations, in this paper, we consider the nonlinear EH model for the SWIPT cooperative communication systems with multiple relays. The piece-wise linear model rather than the conventional linear EH model is used to capture the nonlinear input-output behavior for the energy harvester. Both the TS and PS schemes are considered for the receivers of the relay nodes. We maximize the achievable information rate for the SWIPT multi-relay systems by optimizing the PS or TS ratios for the relay nodes when PS or TS schemes are applied to the EH receivers, respectively. The contributions of the paper are summarized as follows.

(1) For the SWIPT multi-relay cooperative AF systems with TS and PS schemes, we formulate the achievable rate maximization problems as convex and non-convex optimization problems, respectively. For the PS scheme, we transform the non-convex optimization problem into a convex one by transforming the objective function from maximizing the received signal-to-noise ratio (SNR) into minimizing the reciprocal of the received SNR at the destination. To obtain the optimal PS ratios and the maximum achievable rate for the system with PS receiver, the proximal gradient method is used to solve the convex optimization problem that is non-smooth due to the use of the piece-wise linear EH model.
(2) By comparing the performance of the system with linear and nonlinear EH models, the impact of the resource allocation mismatch caused by using the linear EH model in modeling the realistic nonlinear EH circuit is investigated. We also analyze the asymptotic maximum achievable rates at low and high input SNRs for both the TS and PS schemes. It is demonstrated that for the TS scheme, the use of the traditional linear EH model leads to significant resource allocation mismatch and performance degradation, which becomes more serious as the input SNR increases, while for the PS scheme with the optimal PS ratios, the nonlinear EH model obtains only inconspicuous performance gain over the traditional linear EH model, especially in low and high input SNRs region, implying that the use of linear EH model in modeling the realistic nonlinear EH circuit does not bring significant resource allocation mismatch for the PS scheme.
(3) We analyze the effect of the relays’ position and the channel parameters on the system performance. It is shown that farther locations of the relay nodes to the source node bring better achievable rate performance for both of the proposed PS and TS schemes. It is found that for given channel parameters, the performance gap between the linear and nonlinear EH models for the TS scheme is larger than that for the PS scheme, implying that the TS scheme is more susceptible to the resource mismatch than the PS scheme. Meanwhile, the PS scheme is more susceptible to the variation of the relays’ location and the channel parameters than the TS scheme.

The remainder of this paper is organized as follows. In Section 2, the system model is described and the end-to-end SNRs are derived for both the TS and PS receiver. Section 3 provides the optimal problem formulation and solution and Section 4 presents the numerical results and discussion. Finally in Section 5, the paper is concluded.

## 2. System Model

**Figure 1:**
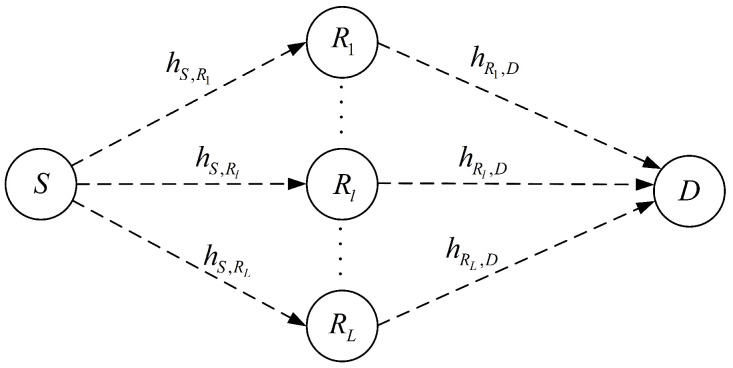
System model.

We consider the multi-relay wireless cooperative system similar to [20,21], where *L* relay nodes assist the source node *S* in transmitting the information data bits to the destination node *D*, as shown in Figure 1. The *L* relay nodes, denoted by a set $ℜ=\left\{R_{1},R_{2},\ldots ,R_{L}\right\}$, are assumed to be energy-limited and capable of harvesting energy from the received signal of *S*. Each relay node uses the harvested energy to forward the source’s information to *D* with AF protocol. It is assumed that there does not exist a direct link between the source and destination nodes due to limited transmit power or shadowing effects [20,21]. Each node is assumed to be equipped with a single antenna and all radio links are assumed to be subjected to independent and frequency non-selective quasi-static fading that the channels keep invariant during the entire communication block with a time duration of *T* [20,31]. The channel coefficients from *S* to the *l*th $R_{l}$ and from $R_{l}$ to *D* are denoted as $h_{S,R_{l}}$ and $h_{R_{l},D}$, respectively. All links are assumed to experience both small-scale Rician fading with Rician factor $K_{R}$ and large-scale path loss effects with path loss exponent β, i.e., the channel coefficients $h_{i,j}$ can be denoted as hi,j=gi,jgi,jdi,jβdi,jβ, where $\left(i,j\right)\in \left\{\left(S,R_{l}\right),\left(R_{l},D\right)\right\}$, $g_{i,j}$ represents the small-scale Rician fading of the channel, and $d_{i,j}$ is the distance between the nodes *i* and *j*. We assume perfect channel state information (CSI) for all of the nodes. In fact, the CSI can be obtained by channel estimation and instantaneous channel feedback, and due to quasistatic fading, the time overhead for the channel estimation from the transmitters to the receivers and the channel feedback from the receivers to the transmitters is negligible compared to the total transmission time [32].

**Figure 2:**
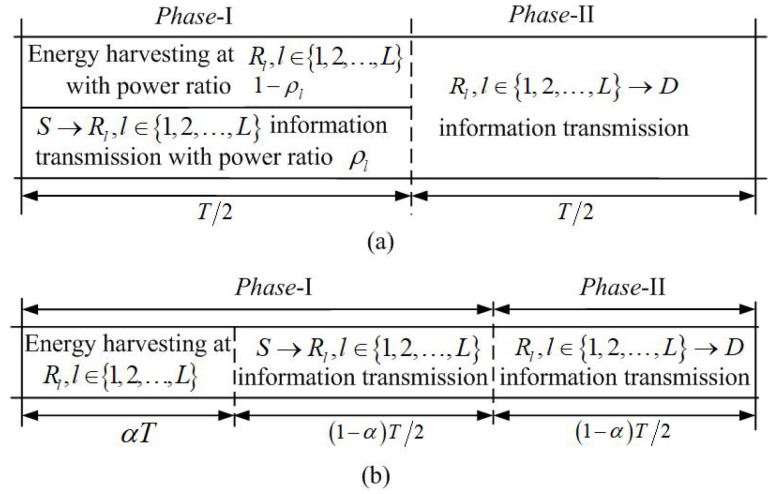
Time slot structure for TS and PS schemes: (**a**) PS scheme, (**b**) TS scheme.

The data transmission of each communication block is divided into two time phases as in [11,12]. A half-duplex mode is applied to the relay nodes for the elimination of the two time phases’s mutual interference. In *phase*-I, the source node broadcasts the information bits while the *L* relay nodes receive the information and harvest the energy with the PS or TS receiver structure. In *phase*-II, *S* keeps silent and the *L* relay nodes amplify the received information signal and forward it to *D* using the energy harvested during *phase*-I. The time slot structure for PS and TS schemes is shown in Figure 2a,b, respectively, where $\rho_{l}$ is the PS ratio for the PS receiver at $R_{l}$ and $\alpha \in \left[0,1\right]$ is the EH time ratio for the TS receiver at all relay nodes. Note that for TS scheme illustrated in Figure 2b, the same EH time ratio is considered for all relay nodes, i.e., $\alpha_{l}=\alpha ,\forall l\in \left\{1,2,\ldots ,L\right\}$, where $\alpha_{l}$ is the EH time ratio of $R_{l}$. The reason for this is that the setting of the same EH time ratio can enhance the efficiency, since different EH time ratios may bring the problem that some relay has to wait until the other relays complete EH [20]. In *phase*-II, we assume that each relay uses a suitable space-time coding, e.g., distributed orthogonal space-time block coding (OSTBC) to achieve diversity gains for the data transmission from the relay nodes to the destination [21,33,34].

We first consider the PS scheme as illustrated in Figure 2a. For the transmission in *phase*-I, the received baseband signal at $R_{l}$ with PS scheme is given as:

(1) $$y_{S,R_{l}}\left(n\right)=\sqrt{P_{s}}h_{S,R_{l}}x_{s}\left(n\right)+N_{R_{l},a}\left(n\right),$$

where $P_{s}$ is the transmit power of the source node *S*, $x_{s}\left(n\right)$ is the information signal transmitted from *S*, and $N_{R_{l},a}\left(n\right)∼CN\left(0,\sigma_{R_{l},a}^{2}\right)$ is the receive antenna noise at $R_{l}$. The received signal $y_{S,R_{l}}\left(n\right)$ at $R_{l}$ is split into two signal streams during *phase*-I. One signal stream is fed into the EH receiver with a power ratio of $1-\rho_{l}$, the other is used for the information decoding with a power ratio of $\rho_{l}$. The signal received at the information receiver (IR) at $R_{l}$ can be expressed as [11,12]:

(2) $$\begin{array}{cc} y_{{\mathrm{IR}}_{l}}\left(n\right) & =\sqrt{\rho_{l}}y_{S,R_{l}}\left(n\right)+N_{R_{l},c}\left(n\right)\\ \; & =\sqrt{\rho_{l}P_{s}}h_{S,R_{l}}x_{s}\left(n\right)+N_{S,R_{l}}\left(n\right), \end{array}$$

where $N_{S,R_{l}}\left(n\right)=\sqrt{\rho_{l}}N_{R_{l},a}\left(n\right)+N_{R_{l},c}\left(n\right)$ is the overall noise at $R_{l}$ and $N_{R_{l},c}\left(n\right)∼CN\left(0,\sigma_{R_{l},c}^{2}\right)$ is the RF to baseband signal conversion noise of $R_{l}$. For the EH receiver of $R_{l}$, the received signal is given by $y_{ER_{l}}^{\mathrm{PS}}\left(n\right)=\sqrt{1-\rho_{l}}y_{S,R_{l}}\left(n\right)$. Therefore, the input RF power at the EH receiver of $R_{l}$ can be derived as $P_{ER_{l}}^{\mathrm{PS}}=\left(1-\rho_{l}\right)P_{s}{\left|h_{S,R_{l}}\right|}^{2}$. When the linear EH model is considered, the energy conversion efficiency of the EH receiver at $R_{l}$ is assumed to be a constant value $\eta_{l}\left(0<\eta_{l}<1\right)$ and the energy harvested at $R_{l}$ is therefore given as [11,25]:

(3) $$P_{EH_{l}}^{\mathrm{PS}}=\eta_{l}P_{ER_{l}}^{\mathrm{PS}}=\eta_{l}\left(1-\rho_{l}\right)P_{s}{\left|h_{S,R_{l}}\right|}^{2}.$$

However, as pointed out before, the linear EH model is inaccurate in modeling realistic nonlinear EH circuit. In fact, it is shown that the output power of the EH receiver first linearly increases then keeps invariant (reaches to saturation) when the input power increases [25]. Hence, here we consider the nonlinear EH model and use the piece-wise linear model that captures the nonlinear saturation characteristic for the EH receiver. The output direct-current (DC) power of the EH receiver at $R_{l}$ is then given by [3,29,31]:

(4) $$P_{EH_{l}}^{\mathrm{PS}}=\min\left\{\eta_{l}P_{ER_{l}}^{\mathrm{PS}},M_{l}\right\},$$

where $M_{l}$ is the maximum (saturation) power that can be harvested at the EH receiver of $R_{l}$ and $\eta_{l}$ is the energy conversion efficiency factor when the EH receiver of $R_{l}$ is not saturated. The power that can be used for the transmission at $R_{l}$ is then given as $P_{R_{l}}^{\mathrm{PS}}=P_{EH_{l}}^{\mathrm{PS}}$.

For *phase*-II transmission, $R_{l}$ first amplifies $y_{{\mathrm{IR}}_{l}}\left(n\right)$, then forwards it to *D*, where the amplification factor $A_{l}$ satisfies the average power constraint and can be denoted as [11,31]:

(5) $$A_{l}=\frac{P_{R_{l}}^{\mathrm{PS}}}{E\left[{\left|y_{\mathrm{IR}}\left(n\right)\right|}^{2}\right]}=\frac{P_{R_{l}}^{\mathrm{PS}}}{\rho_{l}P_{s}{\left|h_{S,R_{l}}\right|}^{2}+\sigma_{S,R_{l}}^{2}},$$

where $\sigma_{S,R_{l}}^{2}$ is the variance of $N_{S,R_{l}}\left(n\right)$ and $E\left[\cdot \right]$ means expectation operation. The signal transmitted from $R_{l}$ is then given as:

(6) $$\begin{array}{cc} x_{R_{l}}\left(n\right) & =\sqrt{A_{l}}y_{IR_{l}}\left(n\right)\\ \; & =\sqrt{A_{l}\rho_{l}P_{s}}h_{S,R_{l}}x_{s}\left(n\right)+\sqrt{A_{l}}N_{S,R_{l}}\left(n\right). \end{array}$$

Since the OSTBC is adopted so that the transmission are mutually orthogonal in time domain for the relays in *phase*-II, the end-to-end SNR at the receiver of the destination *D*, $\gamma^{\mathrm{PS}}$, is equal to the summation of the SNR of all the relay links, i.e., $\gamma^{\mathrm{PS}}=\sum_{l=1}^{L}\gamma_{sr_{l}d}^{\mathrm{PS}}$ [34], where $\gamma_{sr_{l}d}^{\mathrm{PS}}$ is the equivalent instantaneous SNR of the relay link ($S\to R_{l}\to D$). By using the similar way to [11,31,35], $\gamma_{sr_{l}d}^{\mathrm{PS}}$ can be derived as:

(7) $$\gamma_{sr_{l}d}^{\mathrm{PS}}=\frac{A_{l}\rho_{l}P_{s}{\left|h_{S,R_{l}}\right|}^{2}{\left|h_{R_{l},D}\right|}^{2}}{A_{l}{\left|h_{R_{l},D}\right|}^{2}\sigma_{S,R_{l}}^{2}+\sigma_{R_{l},D}^{2}}=\frac{\gamma_{R_{l},D}^{\mathrm{PS}}\gamma_{S,R_{l}}^{\mathrm{PS}}}{\gamma_{R_{l},D}^{\mathrm{PS}}+\gamma_{S,R_{l}}^{\mathrm{PS}}+1},$$

where $\sigma_{R_{l},D}^{2}$ is the variance of the overall noise $N_{R_{l},D}$ (consisting of the receive antenna noise and the RF to baseband signal conversion noise) over the link ($R_{l}\to D$) at *D* and $\gamma_{S,R_{l}}^{\mathrm{PS}}$ and $\gamma_{R_{l},D}^{\mathrm{PS}}$ denote the instantaneous SNRs for the $S\to R_{l}$ and $R_{l}\to D$ links given by, respectively:

(8) γS,RlPS=ρlPshS,Rl21+ρlN01+ρlN022

and:

(9) $$\gamma_{R_{l},D}^{\mathrm{PS}}=\frac{{\left|h_{R_{l},D}\right|}^{2}}{N_{0}}\min\left\{\eta_{l}\left(1-\rho_{l}\right)P_{s}{\left|h_{S,R_{l}}\right|}^{2},M_{l}\right\},$$

where $N_{0}$ is the power spectral density of complex additive white Gaussian noise (AWGN) and it is assumed that for all relay nodes, the power of the receive antenna noise equals to the RF to baseband signal conversion noise, and is the half of the power of total noise at the destination node [12,36], i.e., $\sigma_{R_{l},D}^{2}=2\sigma_{R_{l},a}^{2}=2\sigma_{R_{l},c}^{2}=N_{0},\forall l\in \left\{1,2,\ldots ,L\right\}$. The achievable information rate for the PS scheme can be given as $R^{\mathrm{PS}}=\frac{1}{2}\log_{2}\left(1+\gamma^{\mathrm{PS}}\right)=\frac{1}{2}\log_{2}\left(1+\sum_{l=1}^{L}\gamma_{sr_{l}d}^{\mathrm{PS}}\right)$ [21,33].

We then consider the same TS scheme as [20,37] that is illustrated in Figure 2b, where the transmission time of *phase*-I is divided into two parts. The first part of the transmission time with a duration of αT is used for EH from the source’s RF signal received at $R_{l}$, while the second part of the transmission time with a duration of 1−αT1−αT22 is used for signal receiving at $R_{l}$. For the TS scheme, the signals received at $R_{l}$ in *phase*-I is given as $y_{S,R_{l}}\left(n\right)=\sqrt{P_{s}}h_{S,R_{l}}x_{s}\left(n\right)+N_{R_{l}}\left(n\right)$, where $N_{R_{l}}\left(n\right)∼CN\left(0,\sigma_{R_{l}}^{2}\right)$ is the overall noise consisting of the receive antenna noise and the RF to baseband signal conversion noise at $R_{l}$, $\sigma_{R_{l}}^{2}=N_{0}$. The input RF power and corresponding output DC power of the energy harvester at $R_{l}$ are given as $P_{ER_{l}}^{\mathrm{TS}}=P_{s}{\left|h_{S,R_{l}}\right|}^{2}$ and $P_{EH_{l}}^{\mathrm{TS}}=\min\left\{\eta_{l}P_{ER_{l}}^{\mathrm{TS}},M_{l}\right\}$, respectively. Therefore, the energy harvested by $R_{l}$ during *phase*-I is given as $E_{ER_{l}}^{\mathrm{TS}}=\alpha TP_{EH_{l}}^{\mathrm{TS}}=\alpha T\min\left\{\eta_{l}P_{ER_{l}}^{\mathrm{TS}},M_{l}\right\}$, and the power that can be used for the transmission at $R_{l}$ is then given by:

(10) PRlTS=EERlTS1−αT1−αT22=2α1−αminηlPERlTS,Ml.

During *phase*-II, $R_{l}$ amplifies the received information signal and forwards it to *D* using the harvested energy $P_{R_{l}}^{\mathrm{TS}}$. Similarly to the PS scheme, the equivalent instantaneous SNR of the $l_{th}$ relay link ($S\to R_{l}\to D$) for the TS scheme can be derived as:

(11) $$\gamma_{sr_{l}d}^{\mathrm{TS}}=\frac{\gamma_{R_{l},D}^{\mathrm{TS}}\cdot \gamma_{S,R_{l}}^{\mathrm{TS}}}{\gamma_{R_{l},D}^{\mathrm{TS}}+\gamma_{S,R_{l}}^{\mathrm{TS}}+1},$$

where $\gamma_{S,R_{l}}^{\mathrm{TS}}$ and $\gamma_{R_{l},D}^{\mathrm{TS}}$ represent the instantaneous SNRs of the $S\to R_{l}$ and $R_{l}\to D$ links given by, respectively:

(12) $$\gamma_{S,R_{l}}^{\mathrm{TS}}=\frac{P_{s}{\left|h_{S,R_{l}}\right|}^{2}}{N_{0}}$$

and:

(13) $$\gamma_{R_{l},D}^{\mathrm{TS}}=\frac{{\left|h_{R_{l},D}\right|}^{2}}{N_{0}}\frac{2\alpha }{1-\alpha }\min\left\{\eta_{l}P_{ER_{l}}^{\mathrm{TS}},M_{l}\right\},$$

As illustrated in Figure 2b, the time duration for the information transmission from each of the relay nodes to the destination is 1−αT1−αT22. Hence, the achievable information rate for the TS scheme is $R^{\mathrm{TS}}=\frac{1-\alpha }{2}\log_{2}\left(1+\sum_{l=1}^{L}\gamma_{sr_{l}d}^{\mathrm{TS}}\right)$ [21,33].

## 3. Problem Formulation and Solution

In this section, we consider maximizing the achievable information rate for the SWIPT multi-relay cooperative systems with TS and PS schemes applied to the EH receivers of the relay nodes. Note that for most optimization schemes, maximizing the achievable information rate is a general objective [20,21]. Our goal is to find the optimal PS ratio $\rho_{l},\forall l\in \left\{1,2,\ldots ,L\right\}$ for each relay node when the PS receiver is considered, and the optimal EH time ratio α for all of the relays when the TS receiver is used.

### 3.1. PS Scheme

We first consider the PS scheme. The achievable information rate maximization problem for the PS scheme can be formulated as:

(14) $$\begin{array}{c} \begin{array}{c} \max_{\left\{\rho_{l}\right\}}\;\;R^{\mathrm{PS}}=\frac{1}{2}\log_{2}\left(1+\gamma^{\mathrm{PS}}\right)\\ s.t.\;0\leq \rho_{l}\leq 1,\forall l\in \left\{1,2,\ldots ,L\right\}. \end{array} \end{array}$$

The optimal problem (14) is non-convex, as its objective function is non-concave. Hence, the direct use of the convex optimization techniques cannot solve the problem. Fortunately, it can be found that $\log_{2}\left(1+\gamma^{\mathrm{PS}}\right)$ is a strictly monotonic increasing function of $\gamma^{\mathrm{PS}}$. Therefore, the objective in (14), maximizing the achievable information rate $R^{\mathrm{PS}}=\frac{1}{2}\log_{2}\left(1+\gamma^{\mathrm{PS}}\right)$, is equivalent to maximizing $\gamma^{\mathrm{PS}}=\sum_{l=1}^{L}\gamma_{sr_{l}d}^{\mathrm{PS}}$. Considering that $\rho_{1},\rho_{2},\ldots ,\rho_{L}$ are independent to each other, hence, the summation term $\gamma^{\mathrm{PS}}=\sum_{l=1}^{L}\gamma_{sr_{l}d}^{\mathrm{PS}}$ in the objective function is decomposable, problem (14) can be decomposed into *L* independent sub-problems with identical structure and each can be given as [21]:

(15) $$\begin{array}{c} \begin{array}{c} \max_{\rho_{l}}\;\;\gamma_{sr_{l}d}^{\mathrm{PS}}=\frac{\gamma_{R_{l},D}^{\mathrm{PS}}\cdot \gamma_{S,R_{l}}^{\mathrm{PS}}}{\gamma_{R_{l},D}^{\mathrm{PS}}+\gamma_{S,R_{l}}^{\mathrm{PS}}+1}\\ s.t.\;0\leq \rho_{l}\leq 1. \end{array} \end{array}$$

By substituting (8) and (9) into (15), it is found that (15) is still non-convex. We can solve the maximization problem (15) by minimizing 11γsrldPSγsrldPS subject to the same constraint, namely, transforming (15) into:

(16) $$\begin{array}{c} \begin{array}{c} \min_{\rho_{l}}\;\;\frac{1}{\gamma_{sr_{l}d}^{\mathrm{PS}}}=\frac{1}{\gamma_{S,R_{l}}^{\mathrm{PS}}}+\frac{1}{\gamma_{R_{l},D}^{\mathrm{PS}}}+\frac{1}{\gamma_{R_{l},D}^{\mathrm{PS}}\cdot \gamma_{S,R_{l}}^{\mathrm{PS}}}\\ s.t.\;0<\rho_{l}<1 \end{array}. \end{array}$$

Note that $\rho_{l}=0$ and $\rho_{l}=1$ are excluded from the constraint of (16) to ensure that the denominators of the objective function in (16) will not be zero. Let $\rho_{l}^{*}$ and ${\hat{\rho }}_{l}^{*}$ be the optimal solutions of (15) and (16), respectively. Then, $\rho_{l}^{*}$ can be chosen from ${\hat{\rho }}_{l}^{*}$, 0, and 1 for maximizing $\gamma_{sr_{l}d}^{\mathrm{PS}}$. We will prove that the optimal problem (16) is convex as follows.

Let:

(17) C1,l=2PshS,Rl22PshS,Rl2N0N0,

(18) C2,l=ηlPshS,Rl2hRl,D2ηPshS,Rl2hRl,D2N0N0,

(19) C3,l=MlhRl,D2MlhRl,D2N0N0.

From (8), the first term of the objective function in (16) is given as 11γS,RlPSγS,RlPS=1+11ρlρl1+11ρlρlC1,lC1,l. It can be easily shown that for $0<\rho_{l}<1$, 11γS,RlPSγS,RlPS is a strictly convex function of $\rho_{l}$. The reason for this is that the second derivative of f1(x)=1+11xx with respect to *x* equals to f1′′(x)=22x3x3, which is positive in $x\in \left(0,1\right)$. For the second term of the objective function in (16), 11γRl,DPSγRl,DPS, we first prove that $\gamma_{R_{l},D}^{\mathrm{PS}}$ is concave with respect to $\rho_{l}$ in $\rho_{l}\in \left(0,1\right)$. From (9), $\gamma_{R_{l},D}^{\mathrm{PS}}$ can be rewritten as $\gamma_{R_{l},D}^{\mathrm{PS}}=\min\left\{C_{2,l}\left(1-\rho_{l}\right),C_{3,l}\right\}$. Obviously, $\gamma_{R_{l},D}^{\mathrm{PS}}$ is concave since it is the pointwise minimum of linear functions $C_{2,l}\left(1-\rho_{l}\right)$ and $C_{3,l}$ with respect to $\rho_{l}$. Since $\gamma_{R_{l},D}^{\mathrm{PS}}$ is concave and positive, 11γRl,DPSγRl,DPS is convex [38]. Similarly, for the third term of the objective function in (16), 11γS,RlPSγRl,DPSγS,RlPSγRl,DPS, we first prove that $\gamma_{S,R_{l}}^{\mathrm{PS}}\gamma_{R_{l},D}^{\mathrm{PS}}$ is concave with respect to $\rho_{l}$ in $\rho_{l}\in \left(0,1\right)$. From (8) and (9), $\gamma_{S,R_{l}}^{\mathrm{PS}}\gamma_{R_{l},D}^{\mathrm{PS}}$ can be expressed as:

(20) $$\gamma_{S,R_{l}}^{\mathrm{PS}}\gamma_{R_{l},D}^{\mathrm{PS}}=\min\left\{C_{1,l}C_{2,l}\frac{\rho_{l}\left(1-\rho_{l}\right)}{1+\rho_{l}},C_{1,l}C_{3,l}\frac{\rho_{l}}{1+\rho_{l}}\right\}.$$

Let $f_{2}\left(x\right)=\frac{x\left(1-x\right)}{1+x}$ and $f_{3}\left(x\right)=\frac{x}{1+x}$. It is readily found that ${f_{2}}^{\prime \prime }\left(x\right)<0$ and ${f_{3}}^{\prime \prime }\left(x\right)<0$ for all $x\in \left(0,1\right)$, where ${f_{2}}^{\prime \prime }\left(x\right)$ and ${f_{3}}^{\prime \prime }\left(x\right)$ are the second derivatives of $f_{2}\left(x\right)$ and $f_{3}\left(x\right)$, respectively. Therefore, both $f_{2}\left(x\right)$ and $f_{3}\left(x\right)$ are concave with respect to *x* in $x\in \left(0,1\right)$. This means that $\gamma_{S,R_{l}}^{\mathrm{PS}}\gamma_{R_{l},D}^{\mathrm{PS}}$ is the pointwise minimum of two concave functions C1,lC2,lρl1−ρlρl1−ρl1+ρl1+ρl and C1,lC3,lρlC1,lC3,lρl1+ρl1+ρl with respect to $\rho_{l}$, and hence, they are concave, such that 11γS,RlPSγRl,DPSγS,RlPSγRl,DPS is convex. Since all 11γS,RlPSγS,RlPS, 11γRl,DPSγRl,DPS, and 11γS,RlPSγRl,DPSγS,RlPSγRl,DPS are convex, their sum is convex and problem (16) is convex.

Although (16) is convex, it is non-smooth hence cannot be solved using the standard convex tools. Here, we use the proximal gradient method [39,40] to solve (16). Let f0ρl=11γS,RlPSγS,RlPS=1+11ρlρl1+11ρlρlC1,lC1,l and g0ρl=11γRl,DPSγRl,DPS+11γS,RlPSγRl,DPSγS,RlPSγRl,DPS. Then, the problem (16) can be rewritten as:

(21) $$\begin{array}{c} \begin{array}{c} \min_{\rho_{l}}\;\;f_{0}\left(\rho_{l}\right)+g_{0}\left(\rho_{l}\right)\\ s.t.\;0<\rho_{l}<1 \end{array}. \end{array}$$

Let $h\left(\rho_{l}\right)=f\left(\rho_{l}\right)+g\left(\rho_{l}\right)$. By using the logarithmic barrier method, the inequality constrained minimization problem (21) can be transformed into the unconstrained minimization problem given as:

(22) $$\min_{x}\;h\left(x\right)=f\left(x\right)+g\left(x\right),$$

where we use *x* to denote $\rho_{l}$ for representation convenience, $f\left(x\right)=tf_{0}\left(x\right)-\log x\left(1-x\right)$, $g\left(x\right)=tg_{0}\left(x\right)$, where t>0 is the parameter that sets the accuracy of the logarithmic barrier method [38]. The iterative algorithm based on Beck and Teboulle proximal gradient method [39,40] for solving (22) is given in Algorithm 1 as follows, where $\nabla f\left(x^{\left(l\right)}\right)$ is the gradient of $f\left(x\right)$ at $x^{\left(l\right)}$ and $\mathrm{pro}x_{\lambda g}\left(\cdot \right)$ is the proximal operator of the scaled function λg defined by:

(23) $${\mathrm{prox}}_{\lambda^{\left(l\right)}g}\left(x\right)=\underset{z}{\arg\min}\left(g\left(x\right)+\frac{1}{2\lambda^{\left(l\right)}}{∥z-x∥}_{2}^{2}\right),$$

where λ>0 is the step size that controls the extent to which the proximal operator maps points towards the minimum of *g* [39], ${∥\cdot ∥}_{2}$ is the usual Euclidean norm, ${\hat{f}}_{\lambda^{\left(l\right)}}\left(z,x^{\left(l\right)}\right)$ is given by:

(24) $$\begin{array}{c} {\hat{f}}_{\lambda }\left(z,x^{\left(l\right)}\right)=f\left(x^{\left(l\right)}\right)+\nabla f{\left(x^{\left(l\right)}\right)}^{T}\left(z-x^{\left(l\right)}\right)+\frac{1}{2\lambda }{∥z-x^{\left(l\right)}∥}_{2}^{2}. \end{array}$$

**Remark** **1.** *Complexity and optimality of Algorithm 1. Since the proximal gradient decent satisfies hxl−hx∗≤x0−x∗22x0−x∗222λl2λl for both fixed step size λ and backtracking step size $\lambda^{\left(l\right)}=\beta \lambda^{\left(l-1\right)}$, the computational complexity or the convergence rate of Algorithm 1 is O(1/l) or O(1/ϵ), which also means that an order of 1/ϵ iterations is required to obtain an ϵ-optimal solution for problem (22) [40,41]. Moreover, it can be proven that the generated sequence ${\left\{x^{l}\right\}}_{l\geq 0}$ in Algorithm 1 converges to an optimal solution $x^{*}$ for problem (22) [41].*

**Algorithm 1** Optimization of (22) with Proximal Gradient Method1: **Initialize:**l←0, λ←1, t>0, $\beta \in \left(0,1\right)$, μ>1, $x^{\left(0\right)}\in \left(0,1\right)$2: 
**repeat**
3:     λ:=βλ, t:=μt4:     $z:={\mathrm{prox}}_{\lambda g}\left(x^{\left(l\right)}-\lambda \nabla f\left(x^{\left(l\right)}\right)\right)$5:     l=l+16:     $x^{\left(l\right)}:=z$7: 
**until**

$f\left(z\right)\leq {\hat{f}}_{\lambda }\left(z,x^{\left(l\right)}\right)$



### 3.2. TS Scheme

Let $\gamma^{\mathrm{TS}}=\sum_{l=1}^{L}\gamma_{sr_{l}d}^{\mathrm{TS}}$. To maximize the achievable information rate $R^{\mathrm{TS}}$ of the TS scheme, the optimal problem is then formulated as:

(25) $$\begin{array}{c} \begin{array}{c} \max_{\alpha }\;\;R^{\mathrm{TS}}=\frac{1-\alpha }{2}\log_{2}\left(1+\gamma^{TS}\right)\\ s.t.\;0\leq \alpha \leq 1 \end{array}. \end{array}$$

It can be proven that the objective function in (25) is concave (see Appendix A for detailed proof) so that the problem (25) is convex. By solving (25) using standard convex tools, e.g., the interior point method [38], the optimal EH time ratio α for the relays with TS scheme, $\alpha^{*}$ can be obtained.

### 3.3. Asymptotic Analysis for the Maximum Achievable End-to-End Rate

In this section, in order to get more intuitive insight about the effect of nonlinear EH model on achievable information rate maximization for the system with TS and PS schemes, we analyze the asymptotic maximum achievable information rate for the system with nonlinear EH model in the region of low and high input SNRs, say $P_{s}\to 0$ and $P_{s}\to \infty$ with fixed noise power $N_{0}$ and saturation harvested power $M_{l}$, which is then compared with traditional linear EH model so as to further investigate the impact of the resource allocation mismatch caused by using a traditional linear EH model.

#### 3.3.1. Asymptotic Analysis for the PS Scheme

By substituting (17)–(19) into (7), $\gamma_{sr_{l}d}^{\mathrm{PS}}$ can be rewritten as:

(26) $$\gamma_{sr_{l}d}^{\mathrm{PS}}=\frac{C_{1,l}\frac{\rho_{l}}{1+\rho_{l}}\min\left\{C_{2,l}\left(1-\rho_{l}\right),C_{3,l}\right\}}{C_{1,l}\frac{\rho_{l}}{1+\rho_{l}}+\min\left\{C_{2,l}\left(1-\rho_{l}\right),C_{3,l}\right\}+1}.$$

From (14) and (26), the practical maximum achievable information rate is given by:

(27) $$\begin{array}{c} R_{\max-\mathrm{PS}}=\frac{1}{2}\log_{2}\left(1+\sum_{l=1}^{L}\frac{C_{1,l}\frac{\rho_{l}^{*}}{1+\rho_{l}^{*}}\min\left\{C_{2,l}\left(1-\rho_{l}^{*}\right),C_{3,l}\right\}}{C_{1,l}\frac{\rho_{l}^{*}}{1+\rho_{l}^{*}}+\min\left\{C_{2,l}\left(1-\rho_{l}^{*}\right),C_{3,l}\right\}+1}\right) \end{array}.$$

Let $x_{l}^{*}$ and $y_{l}^{*}$ be the optimal PS ratio $\rho_{l}^{*}$ obtained for relay $R_{l}$ with the linear and nonlinear EH models, respectively. Since the practical EH circuits at the relay nodes are nonlinear, the practical maximum achievable information rate is given as (27) whether the linear and nonlinear EH models are used. Then, from (27), by substituting $\rho_{l}^{*}$ with $x_{l}^{*}$ and $y_{l}^{*}$, the practical maximum achievable information rate, when the linear and nonlinear EH models are used, can be, respectively, expressed as:

(28) $$\begin{array}{c} R_{\max-\mathrm{PS}}^{linear}=\frac{1}{2}\log_{2}\left(1+\sum_{l=1}^{L}\frac{C_{1,l}\frac{x_{l}^{*}}{1+x_{l}^{*}}\min\left\{C_{2,l}\left(1-x_{l}^{*}\right),C_{3,l}\right\}}{C_{1,l}\frac{x_{l}^{*}}{1+x_{l}^{*}}+\min\left\{C_{2,l}\left(1-x_{l}^{*}\right),C_{3,l}\right\}+1}\right) \end{array}$$

and:

(29) $$\begin{array}{c} R_{\max-\mathrm{PS}}^{nonlinear}=\frac{1}{2}\log_{2}\left(1+\sum_{l=1}^{L}\frac{C_{1,l}\frac{y_{l}^{*}}{1+y_{l}^{*}}\min\left\{C_{2,l}\left(1-y_{l}^{*}\right),C_{3,l}\right\}}{C_{1,l}\frac{y_{l}^{*}}{1+y_{l}^{*}}+\min\left\{C_{2,l}\left(1-y_{l}^{*}\right),C_{3,l}\right\}+1}\right) \end{array}.$$

Case 1 (Low Input SNR) with $P_{s}\to 0$: When $P_{s}\to 0$, it can be obtained from (17) that $C_{1,l}\to 0$. Then, from (28) and (29), the practical maximum achievable information rate is equal to zero whether the nonlinear or linear EH model is used for the EH receivers at the relay nodes, which means that the maximum achievable rate is the same for both EH models when $P_{s}\to 0$. Actually, when $P_{s}$ is so small that $C_{2,l}\ll C_{3,l}$ and $\min\left\{C_{2,l}\left(1-\rho_{l}\right),C_{3,l}\right\}=C_{2,l}\left(1-\rho_{l}\right)$, i.e., the EH receivers have small input power and are not saturated, the nonlinear EH model has the same effect on system performance as the linear EH model. In this case, the nonlinear EH model is essentially equivalent to traditional linear one, which results in the same optimal PS ratios $x_{l}^{*}$ and $y_{l}^{*}$ and, therefore, brings the same system performance for both EH models.

Case 2 (High Input SNR) with $P_{s}\to \infty$: when $P_{s}\to \infty$, it can be obtained from (17) and (18) that $C_{1,l}\to \infty$ and $C_{2,l}\to \infty$ such that $C_{2,l}\gg C_{3,l}$ and $\min\left\{C_{2,l}\left(1-\rho_{l}\right),C_{3,l}\right\}=C_{3,l}$. Then, from (28) and (29), the practical maximum achievable information rate, when the linear or nonlinear EH models are used, can be derived as $R_{\max-\mathrm{PS}}^{linear}\approx \frac{1}{2}\log_{2}\left(1+\sum_{l=1}^{L}C_{3,l}\right)$ and $R_{\max-\mathrm{PS}}^{nonlinear}\approx \frac{1}{2}\log_{2}\left(1+\sum_{l=1}^{L}C_{3,l}\right)$, which means that for high SNR, the maximum achievable rate is the same for both EH models.

#### 3.3.2. Asymptotic Analysis for the TS Scheme

Let $\alpha^{*}$ be the optimal TS ratio. Then, from (11) and (25), the maximum achievable information rate can be expressed as:

(30) $$\begin{array}{c} R_{\max-\mathrm{TS}}=\frac{1-\alpha^{*}}{2}\log_{2}\left(1+\sum_{l=1}^{L}\frac{2\alpha^{*}C_{4,l}C_{5,l}}{2\alpha^{*}C_{4,l}+\left(C_{5,l}+1\right)\left(1-\alpha^{*}\right)}\right) \end{array},$$

where $C_{4,l}$ and $C_{5,l}$ are given as (36) and (37) in Appendix, respectively.

Case 1 (Low Input SNR) with $P_{s}\to 0$: when $P_{s}\to 0$, it can be obtained from (37) that $C_{5,l}\to 0$. Then, from (30), the practical maximum achievable information rate is equal to zero for both the nonlinear and linear EH models. Similarly to the PS scheme, when $P_{s}$ is so small that the input RF power does not saturate the EH receiver, the same optimal TS ratios can be obtained, leading to the same maximum achievable information rate for both the linear and nonlinear EH models.

Case 2 (High Input SNR) with $P_{s}\to \infty$: When $P_{s}\to \infty$, it can be obtained from (19), (36) and (37) that $C_{4,l}=C_{3,l}$ and $C_{5,l}\to \infty$. Then from (30), the practical maximum achievable information rate can be expressed as:

(31) $$R_{\max-\mathrm{TS}}=\frac{1-\alpha^{*}}{2}\log_{2}\left(1+\sum_{l=1}^{L}\frac{2\alpha^{*}}{1-\alpha^{*}}C_{3,l}\right).$$

Let $x^{*}$ and $y^{*}$ be the optimal TS ratios $\alpha^{*}$ when the linear and nonlinear EH models are used, which can be obtained by solving the optimal problem (25) and given as:

(32) $$\begin{array}{c} x^{*}=\underset{0\leq x\leq 1}{\arg\min}\frac{x-1}{2}\log_{2}\left(1+\sum_{l=1}^{L}\frac{2xC_{4,l}^{linear}C_{5,l}}{2xC_{4,l}^{linear}+\left(C_{5,l}+1\right)\left(1-x\right)}\right) \end{array}$$

and:

(33) $$\begin{array}{c} y^{*}=\underset{0\leq y\leq 1}{\arg\min}\frac{y-1}{2}\log_{2}\left(1+\sum_{l=1}^{L}\frac{2yC_{4,l}C_{5,l}}{2yC_{4,l}+\left(C_{5,l}+1\right)\left(1-y\right)}\right), \end{array}$$

where C4,llinear=ηlPshS,Rl2hRl,D2ηlPshS,Rl2hRl,D2N0N0. When $P_{s}\to \infty$, it can be obtained that $C_{4,l}^{linear}\to \infty$, $C_{5,l}\to \infty$, and C4,l=MlhRl,D2MlhRl,D2N0N0≪C5,l. Then, (32) and (33), respectively, can be rewritten as:

(34) $$x^{*}=\underset{0\leq x\leq 1}{\arg\min}\frac{x-1}{2}\log_{2}\left(1+\sum_{l=1}^{L}C_{5,l}\right)=0$$

and:

(35) $$y^{*}=\underset{0\leq y\leq 1}{\arg\min}\frac{y-1}{2}\log_{2}\left(1+\frac{2y}{1-y}\sum_{l=1}^{L}\frac{M_{l}{\left|h_{R_{l},D}\right|}^{2}}{N_{0}}\right),$$

Let $R_{\max-\mathrm{TS}}^{linear}$ and $R_{\max-\mathrm{TS}}^{nonlinear}$ be the practical maximum achievable information rates for the linear and nonlinear EH models, which can be obtained from (31) by substituting $\alpha^{*}$ with $x^{*}$ and $y^{*}$, respectively. From (34) and (35), it can be obtained that when $P_{s}\to \infty$, $x^{*}\to 0$ and thus $R_{\max-\mathrm{TS}}^{linear}\to 0$, whereas $y^{*}$ does not changes with $P_{s}$ such that $R_{\max-\mathrm{TS}}^{nonlinear}$ is invariable when $P_{s}$ changes.

## 4. Numerical Results

In this section, numerical results are presented to demonstrate the performance of the SWIPT multi-relay cooperative system with nonlinear EH model for the proposed PS scheme (14) and TS scheme (25). For comparison, the numerical results for the system with traditional linear EH model are also depicted. With the linear EH model, for the PS scheme (14), the energy harvested at the relay nodes is given as (3) rather than (4), while for the TS scheme (25), the power that can be used for the transmission at $R_{l}$ is given as $P_{R_{l}}^{\mathrm{TS}}=\frac{2\alpha }{1-\alpha }\eta_{l}P_{ER_{l}}^{\mathrm{TS}}$ rather than (10). We consider that all nodes are located in a two-dimensional plane, and that the *L* relay nodes are randomly but uniformly distributed within a circle (called the relay nodes circle) with a radius of 1 meter. The center of the circle is located on the straight line between *S* and *D*. The distance between *S* and the center of the relay nodes circle, and between the center of the relay nodes circle and *D*, are denoted as $d_{sc}$ and $d_{cd}$, respectively. Unless otherwise specified, the simulation parameters for the numerical examples are set as follows. The number of the relay nodes is equal to 5. The transmission power of *S* is set to $P_{s}=30$ dBm. For each relay node $R_{l},\forall l\in \left\{1,2,\ldots ,L\right\}$, the harvested saturation power $M_{l}$ and the energy conversion efficiency factor $\eta_{l}$ when the EH circuit is not saturated, is assume to be the same and set to $M_{l}=24$ mW and $\eta_{l}=0.7$ as in [25,31], respectively. The results in this section are obtained by averaging over 1000 channel including small-scale Rician fading and large-scale path loss realizations.

**Figure 3:**
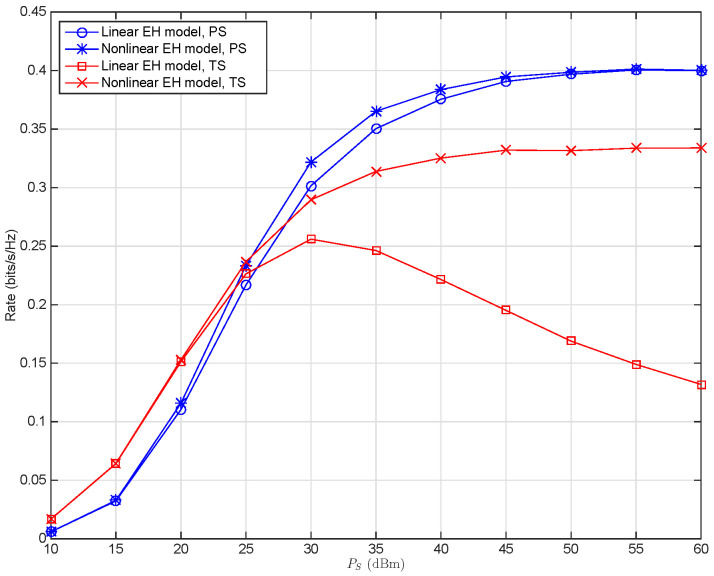
Maximum achievable rate versus the transmit power $P_{s}$ (dBm) for the SWIPT multi-relay cooperative system with PS and TS schemes when $N_{0}=10$ dBm, $K_{R}=0$ dB, β=2, $d_{sc}=2$ m, $d_{cd}=4$ m.

In Figure 3, the maximum achievable rate is depicted for the SWIPT wireless multi-relay systems with various transmit power $P_{s}$. It can be observed that when the nonlinear EH model is used for the optimization, for larger input SNR, the PS scheme outperforms the TS scheme, whereas for smaller input SNR, the opposite result can be observed, where the input SNR is denoted as $P_{s}/N_{0}$. This observation is the same as the results in [21,37] for the SWIPT relay systems with the linear EH model. It shows that for the nonlinear EH model, both the maximum achievable rates of the system with the TS and PS schemes monotonically increase with the transmit power $P_{s}$, whereas when the linear EH model is applied to the system, for the PS scheme, the maximum achievable rate is still a monotonically increasing function with respect to $P_{s}$, while for the TS scheme, it is a concave function of $P_{s}$. From another perspective, the use of the linear EH model leads to different degrees of performance degradation for the TS and PS schemes. Specifically, the nonlinear EH model is significantly superior to the linear EH model in the system performance for the proposed TS scheme in the region of both medium and high SNRs, whereas it slightly outperforms the linear EH model for the PS scheme only in the range of medium SNR. Compared with the nonlinear EH model, the system performance degradation of the linear EH model is due to the resource allocation mismatch, which is caused by using the linear EH model, while it does not account for the nonlinear characteristic of the EH circuits and the saturation of the input RF power [25].

**Remark** **2.** *Asymptotic Performance of the nonlinear EH model. It can be observed in Figure 3 that in the low input SNR region, the system performance of the nonlinear EH model is same as that of the linear EH model for both the TS and PS schemes, while in the region of high input SNR, the system performance of the nonlinear EH model, compared to that of the linear EH model, is also the same for the PS scheme, whereas it is much different for the TS scheme. The reason is that, as analyzed in Section 3, low input SNR makes the nonlinear EH model equivalent to the linear one, which results in the same optimal PS ratio $\rho_{l}^{*}$ or TS ratio $\alpha^{*}$, and therefore, brings the same system performance for nonlinear and linear EH models, and that in the region of high input SNR, for the PS scheme, the practical maximum achievable rate of the system is the same and tends to be invariable for both EH models, whereas for the TS scheme, it tends to be zero for the linear EH model and invariable for the nonlinear EH model.*

**Remark** **3.** *Resource allocation mismatch of the linear EH model. The performance of the system with the linear EH model, compared to that with the nonlinear EH model, is slightly changed for the PS scheme, whereas it significantly declined for the TS scheme, implying that the impact of the resource allocation mismatch brought by the use of traditional linear EH model is inconspicuous for the PS scheme, whereas this is much more serious for the TS scheme, especially in higher SNRs. Specifically, it is shown in Figure 3 that in the region of high SNR, the maximum achievable rate for the TS scheme even decreases for the linear EH model while keeps invariant for the nonlinear EH model when the SNR increases. The reason is that, as analyzed in Section 3, for high input SNR, the optimal TS ratio $y^{*}$ for the nonlinear EH model keeps invariable as $P_{s}$ changes, whereas the optimal TS ratio $x^{*}$ for linear EH model gets smaller when the SNR increases ($x^{*}\to 0$ when $P_{s}\to \infty$), as shown in Table 1. From (31), in the region of high input SNR, the decreasing optimal TS ratio $x^{*}$ will lead to a more rapid decrease in the received SNR at D denoted as $\frac{2x^{*}}{1-x^{*}}\sum_{l=1}^{L}C_{3,l}$ than the increase in the information transmission time ratio $\frac{1-x^{*}}{2}$, and therefore, leading to the decrease in the practical maximum achievable rate for the system with nonlinear EH circuit.*

**Figure 4:**
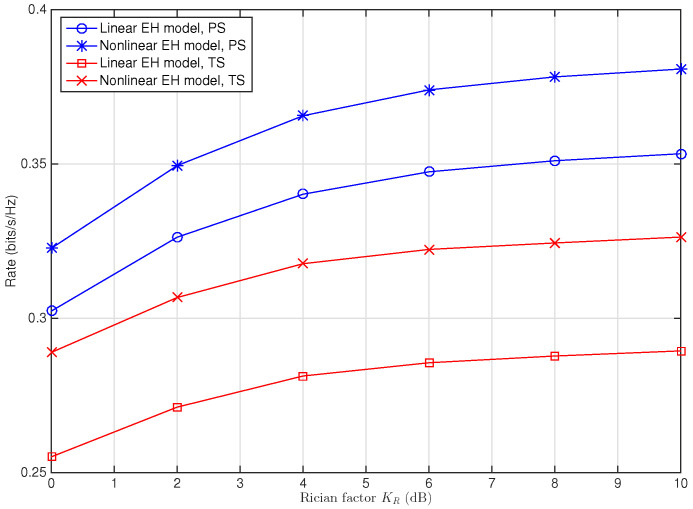
Maximum achievable rate versus the Rician factor $K_{R}$ for SWIPT multi-relay cooperative system with PS and TS schemes when $P_{s}/N_{0}=20$ dB, β=2, $d_{sc}=2$ m, $d_{cd}=4$ m.

Figure 4 shows the impact of variation of Rician factor $K_{R}$ on the maximum achievable rate for the system with a medium level of SNR ($P_{s}/N_{0}=20$ dB). It can be observed that for both the TS and PS schemes, the rate increases as $K_{R}$ increases whether the nonlinear EH model or linear EH model is considered, since a larger Rician factor $K_{R}$ means a better channel condition. It is shown that compared with the conventional linear EH model, both the TS and PS schemes obtain performance gain despite the variation of Rician factor $K_{R}$ when the the nonlinear EH model is adopted. Moreover, the performance gain is more larger for the TS scheme than that for the PS scheme when $K_{R}$ is same. On the other hand, it is shown that the performance gap between the linear and nonlinear EH models does not change much for the TS scheme, while it obviously increases for the PS scheme when $K_{R}$ increases. Specifically, the performance gap increases 35.47% (from 0.0203 bits/s/Hz to 0.0275 bits/s/Hz) for the PS scheme while it increases 9.17% (from 0.0338 bits/s/Hz to 0.0369 bits/s/Hz) for the TS scheme when $K_{R}$ increases from 0 dB to 10 dB.

**Figure 5:**
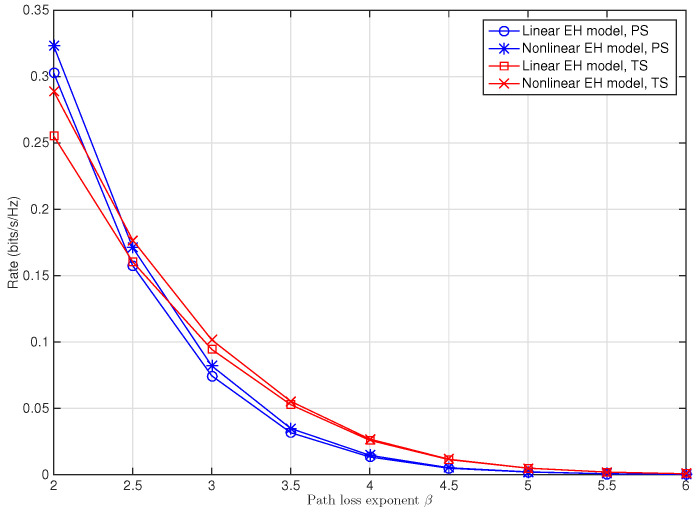
Maximum achievable rate versus the path loss exponent β for SWIPT multi-relay cooperative system with PS and TS schemes when $P_{s}/N_{0}=20$ dB, $K_{R}=0$ dB, $d_{sc}=2$ m, $d_{cd}=4$ m.

In Figure 5, the maximum achievable rate is plotted against the path loss exponent β. Not surprisingly, it is shown that the rate decreases rapidly as β increases for both PS and TS schemes, since a larger path loss exponent means a much more transmission loss. Moreover, it is shown that the maximum achievable rate for the PS scheme decreases more rapidly than the TS scheme when β increases, which means that the PS scheme is more susceptible to the variation of path loss exponent. As for the performance degradation due to the resource allocation mismatch brought by using linear EH model instead of the nonlinear one, it is demonstrated that for the same value of β, the performance gap between the linear EH and nonlinear EH models for the TS scheme is more larger than that for the PS scheme, and that when β gets larger, the gap becomes smaller until it disappears for both of the TS and PS schemes. The reason is that the larger path loss exponent brings much more transmission loss and means smaller SNR, so that the input RF power dose not saturate the EH receiver. As mentioned before, this leads to the same optimal PS ratio $\rho_{l}^{*}$ or TS ratio $\alpha^{*}$, and thus, brings the same system performance for the nonlinear and linear EH models. It can be observed that for smaller β, the PS scheme outperforms the TS scheme, whereas for larger β, the result is opposite. This is in line with the observation in Figure 3 that for larger SNR, the PS scheme is superior to the TS scheme, whereas for smaller SNR, the TS scheme is superior, since a smaller β means a higher received SNR.

**Figure 6:**
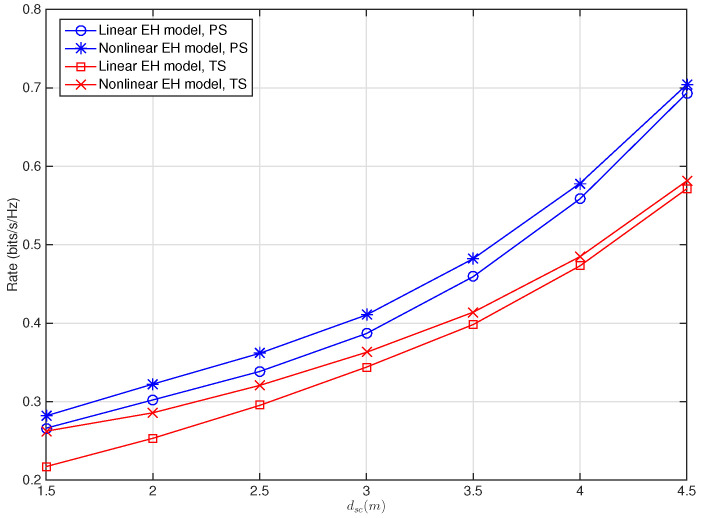
Maximum achievable rate versus $d_{sc}$ for SWIPT multi-relay cooperative system with PS and TS schemes when $P_{s}/N_{0}=20$ dB, $K_{R}=0$ dB, β=2, $d_{sc}+d_{cd}=6$ m.

Figure 6 demonstrates the impact of the location of the relay nodes denoted by $d_{sc}$ on the maximum achievable rate. It can be observed that the performance gets worse as $d_{sc}$ decreases for both the PS and TS schemes. This observation is aligned with that in [31], where single relay with nonlinear EH model is considered for the SWIPT relay systems. It is also shown that the PS scheme is more susceptible to the variation of $d_{sc}$ than the TS scheme, i.e., the maximum achievable rate for the PS scheme increases more rapidly than the TS scheme when $d_{sc}$ increases, whereas the performance gap between the linear and nonlinear EH models for the TS scheme is larger than that for the PS scheme, especially for smaller $d_{sc}$.

**Remark** **4.** *Channel susceptibility of the PS and TS schemes. Figure 3, Figure 4, Figure 5 and Figure 6 show that, for given channel parameters Rician factor $K_{R}$, path loss exponent β, and relays’ location denoted by $d_{sc}$, using a linear EH model in modeling the practical EH circuit brings larger performance degradation for the system with the TS scheme than that with the PS scheme, implying that the TS scheme is more susceptible to the resource allocation mismatch caused by using the linear EH model than the PS scheme. Conversely, for the variation of the relays’ location, Rician factor $K_{R}$, and path loss exponent β, more rapid performance change can be observed for the PS scheme, implying that the PS scheme is more susceptible to the variation of the channels than the TS scheme.*

## 5. Conclusions

In this paper, we have proposed the optimal TS and PS schemes for the maximization of the achievable rate for the SWIPT multi-relay cooperative AF communication system with a nonlinear EH model. The impact of the resource allocation mismatch due to using the traditional linear EH model in modeling realistic nonlinear EH circuit on the performance, has been investigated for both of the proposed TS and PS schemes. The effect of the relays’ position and the channel parameters on the system performance has also been investigated. Simulation results have demonstrated that the use of the linear EH model in modeling the practical EH circuit for the resource allocation brings significant performance degradation for the TS scheme, whereas inconspicuous performance change was observed for the PS scheme. It has also been demonstrated that the TS scheme is more susceptible to the resource allocation mismatch than the PS scheme for given channel parameters, and that the PS scheme is more susceptible to the variation of the relays’ location and the channel parameters than the TS scheme.

## Figures and Tables

**Table 1:** Optimal TS ratios for linear and nonlinear EH models.

Transmit Power (dBm)	$P_{s}=30$	$P_{s}=35$	$P_{s}=40$	$P_{s}=45$	$P_{s}=50$	$P_{s}=55$	$P_{s}=60$
Optimal TS ratios $x^{*}$ for linear EH model	0.4227	0.3390	0.2734	0.2229	0.1867	0.1584	0.1372
Optimal TS ratios $y^{*}$ for nonlinear EH model	0.6054	0.6012	0.6006	0.5994	0.6008	0.6003	0.6003

## Data Availability

The data presented in this study are available on request from the corresponding author.

## Appendix A

Let:

(A1) C4,l=minηlPERlTS,MlhRl,D2hRl,D2N0N0

and:

(A2) C5,l=PshS,Rl2PshS,Rl2N0N0.

By denoting $\gamma_{sr_{l}d}^{\mathrm{TS}}$ as $f_{l}\left(\alpha \right)$ and substituting (12) and (13) into (11), $f_{l}\left(\alpha \right)$ can be expressed as:

(A3) $$f_{l}\left(\alpha \right)=\frac{2\alpha C_{4,l}C_{5,l}}{2\alpha C_{4,l}+\left(C_{5,l}+1\right)\left(1-\alpha \right)}.$$

By substituting (A3) into the objective function in (25), the second derivative of $R^{\mathrm{TS}}$ with respect to α can be obtained as:

(A4) $$\begin{array}{c} {\left(R^{\mathrm{TS}}\right)}^{\prime \prime }=\frac{\sum_{l=1}^{L}\left[\left(1-\alpha \right){f_{l}}^{\prime \prime }\left(\alpha \right)-2{f_{l}}^{\prime }\left(\alpha \right)\right]}{2\log_{2}\left[1+\sum_{l=1}^{L}f_{l}\left(\alpha \right)\right]}-\frac{\left(1-\alpha \right){\left[\sum_{l=1}^{L}{f_{l}}^{\prime }\left(\alpha \right)\right]}^{2}}{2\log_{2}{\left[1+\sum_{l=1}^{L}f_{l}\left(\alpha \right)\right]}^{2}}, \end{array}$$

where ${f_{l}}^{\prime }\left(\alpha \right)$ and ${f_{l}}^{\prime \prime }\left(\alpha \right)$ are the first and second derivative of $f_{l}\left(\alpha \right)$ with respect to α, respectively. Since $\alpha \in \left(0,1\right)$, it is obviously that the second term in (A4) is negative. For the first term in (A4), it can be derived that:

(A5) $$\begin{array}{c} \left(1-\alpha \right){f_{l}}^{\prime \prime }\left(\alpha \right)-2{f_{l}}^{\prime }\left(\alpha \right)=\frac{-8C_{5,l}\left(C_{5,l}+1\right)C_{4,l}^{2}}{{\left[2\alpha C_{4,l}+\left(C_{5,l}+1\right)\left(1-\alpha \right)\right]}^{3}}. \end{array}$$

Since $C_{4,l}>0$, $C_{5,l}>0$ and $\alpha \in \left(0,1\right)$, it can be easily obtained from (A3) and (A5) that $f_{l}\left(\alpha \right)>0$ and $\left(1-\alpha \right){f_{l}}^{\prime \prime }\left(\alpha \right)-2{f_{l}}^{\prime }\left(\alpha \right)<0$, and hence, the first term in (A4) is negative. Therefore, the second derivative of $R^{\mathrm{TS}}$ with respect to α given by (A4) is negative in $\alpha \in \left(0,1\right)$, which proves that $R^{\mathrm{TS}}$ is the concave function with respect to α in $\alpha \in \left(0,1\right)$.
